# A de novo acute myeloid leukemia (AML-M4) case with a complex karyotype and yet unreported breakpoints

**DOI:** 10.1186/1755-8166-6-18

**Published:** 2013-05-05

**Authors:** Walid Al-achkar, Abdulmunim Aljapawe, Moneeb Abdullah Kassem Othman, Abdulsamad Wafa

**Affiliations:** 1 Department of Molecular Biology and Biotechnology, Human Genetics Division, Atomic Energy Commission, P.O. Box 6091, Damascus, Syria; 2 Department of Molecular Biology and Biotechnology, Mammalians Biology Division, Atomic Energy Commission, Damascus, Syria; 3 Institute of Human Genetics, Jena University Hospital, Jena, Germany

**Keywords:** Acute myeloid leukemia (AML), Chromosomal abnormalities, Fluorescence in situ hybridization (FISH), Array-proven multicolor banding (aMCB)

## Abstract

**Background:**

Acute myelogeneous leukemia (AML) is a malignancy of the hematopoietic stem cells, for which cytogenetic analysis is still one of the most important diagnostic and prognostic tools. Still, we are far away from having seen and described all possible genetic changes associated with this kind of acquired disease.

**Results:**

Bone marrow cells of a female patient with clinical diagnoses of AML and immunophenotypically confirmed AML-M4 were studied by GTG-banding. The later was not able to resolve all karyotypic changes and the complex karyotype was characterized in more detail by fluorescence in situ hybridization (FISH) and array-proven multicolor banding (aMCB). To the best of our knowledge, the present case is the only one ever seen with a del(5)(q14q34), a der(17)t(4;17)(p13;p13), a del(2)(p23), a der(4)t(4;7)(p13;q11.23), a der(22)t(11;22)(q23;q11.2) and two complex rearranged chromosomes 11 involving chromosomes 7 and 22 as well as 2.

**Conclusions:**

The yet unreported breakpoints observed in this case seem to be correlated with an adverse prognosis. Overall, molecular cytogenetic studies are suited best for identification and characterization of chromosomal rearrangements in acute leukemia and single case reports as well as large scale studies are necessary to provide further insides in karyotypic changes taking place in human malignancies.

## Background

Acute myelogeneous leukemia (AML) is a disease of the myeloid compartment of the hematopoietic system and is characterized by the accumulation of undifferentiated blast cells in the peripheral blood and bone marrow [1]. Cytogenetics is considered the most important independent prognostic parameter in AML [2,3]. Chromosomal abnormalities also provide useful information for monitoring residual disease [4]. Most of chromosomal abnormalities are detectable by banding cytogenetic analysis, and they occur in 55% of de novo AML in adults [5,6]. Some chromosomal aberrations in AML are recurrent and closely associated with specific cytomorphological subtypes according to French-American-British (FAB) criteria [7-10]. However, 5-10% of AML patients present with multiple chromosomal rearrangements involving three or more chromosomes. These patients usually have a poor prognosis, and it is likely that some of these rearrangements contribute to their disease progression [2].

We present a primary AML-M4 case with yet unreported translocation events including seven different chromosomes.

## Results

**Figure 1:**
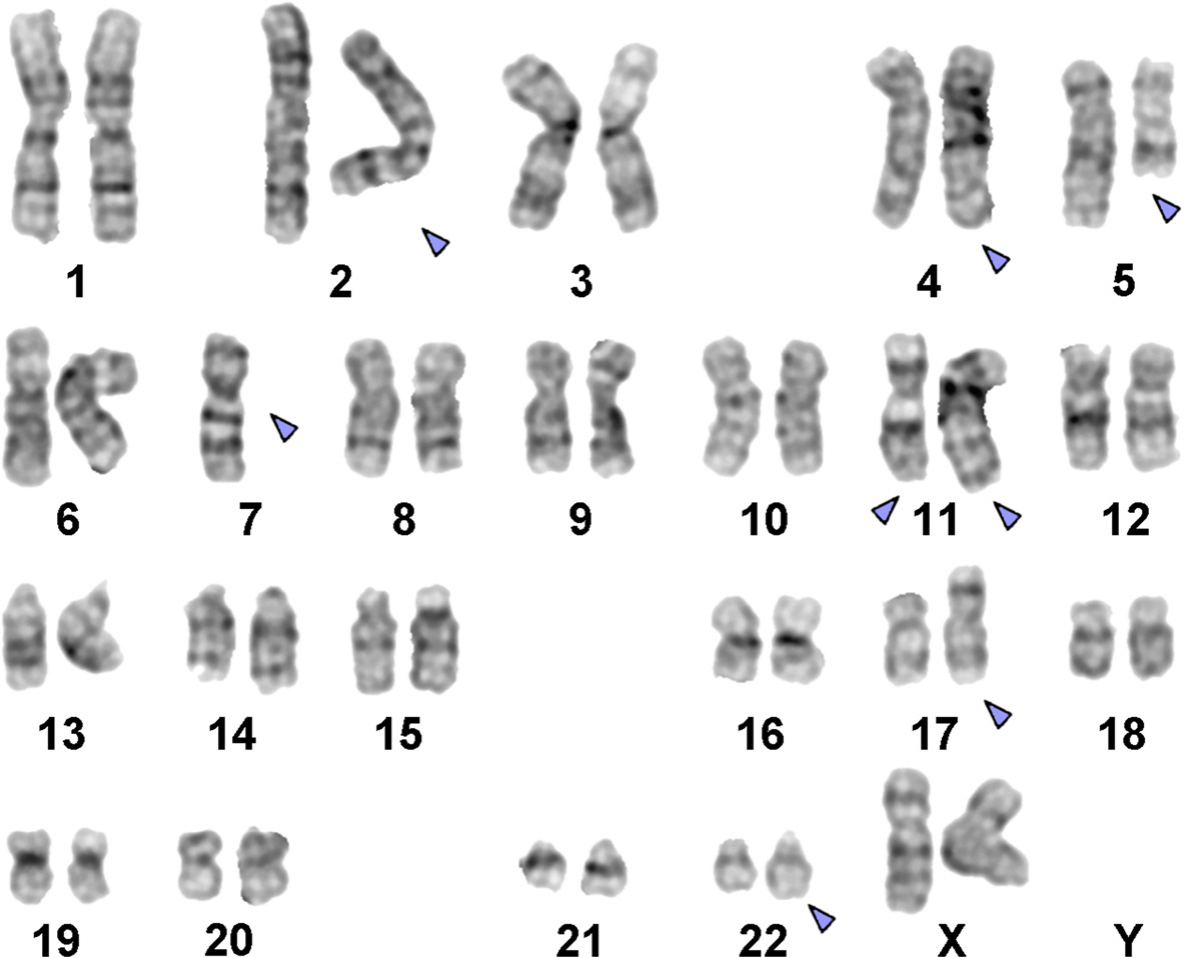
**GTG-banding revealed a complex karyotype involving six chromosomes and monosomy 7.** All derivative or clonally missing chromosomes are highlighted by arrowheads.

**Figure 2:**
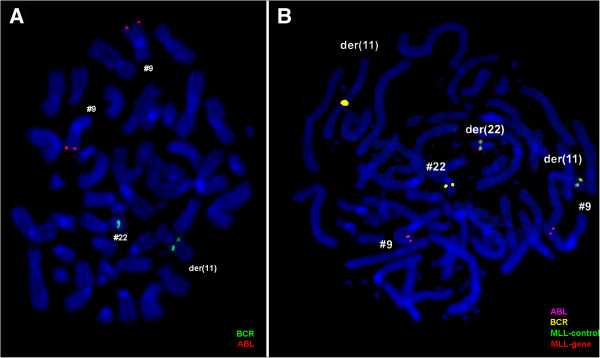
**FISH-results using locus-specific probes.** (**A**) Metaphase FISH using probes for BCR (green) and ABL (orange) showed two orange signals on the two chromosomes 9, one green on the chromosome 22 and the other green signal was observed on der(11). (**B**) Metaphase FISH using probes for BCR (yellow) and ABL (red) mixed with MLL break-apart probe showed one fusion signal was located on the short arm of der(11), the second fusion signal was observed on der(22), two orange signals on the two chromosomes 9, one green on the chromosome 22 and the other green signal was observed on der(11). Abbreviations: # = chromosome; der = derivative chromosome.

**Figure 3:**
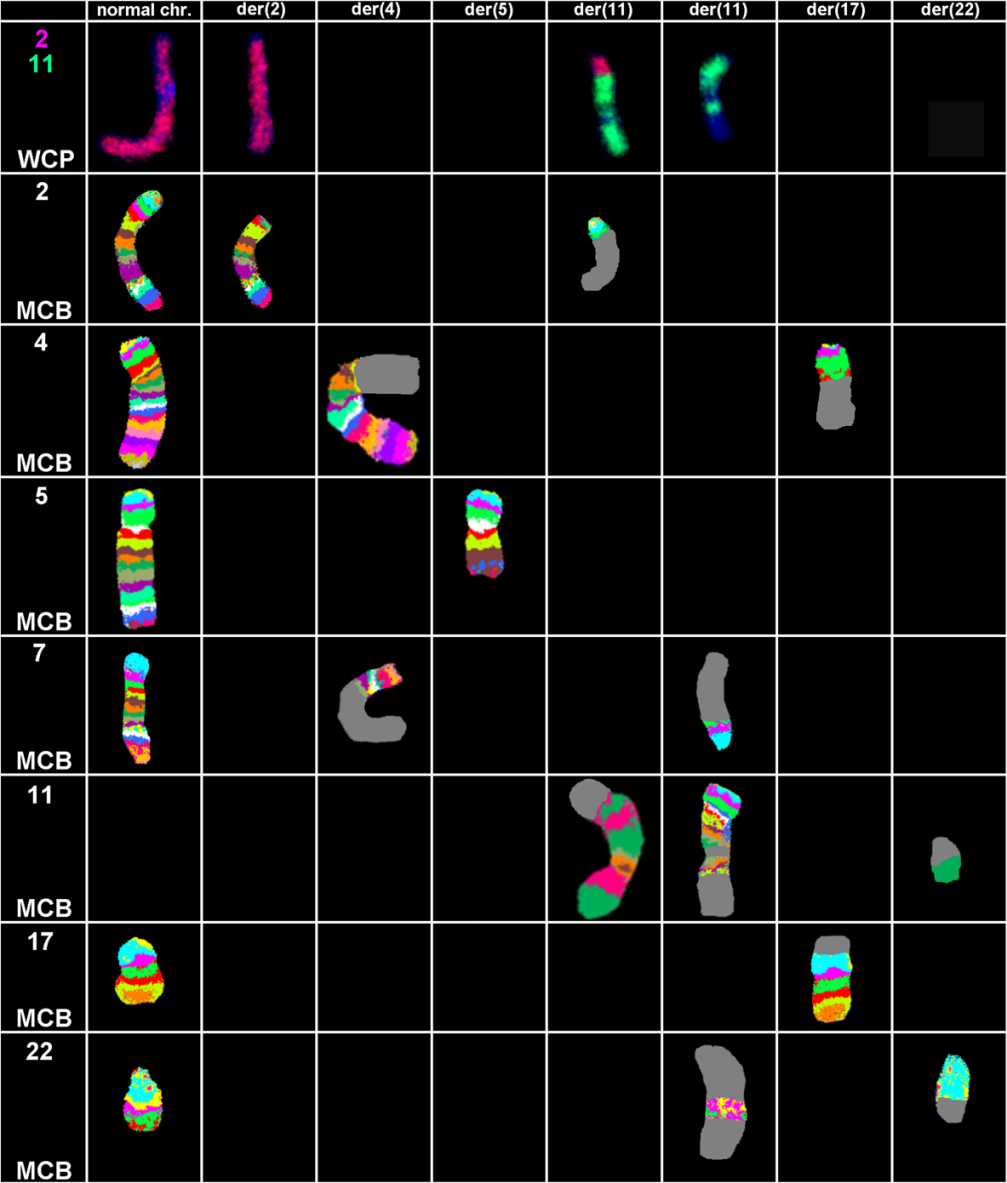
**Array-proven multicolor banding (aMCB) was applied to determine the involved in this complex rearrangement.** In each lane the results of aMCB analysis using probe-sets for chromosomes 2, 4, 5, 7, 11, 17 and 22 are shown. The normal chromosomes are shown in the first column, the derivative of all five chromosomes in the following ones. In the light gray by aMCB-probes unstained regions on the derivative chromosomes are depicted. Abbreviations: # = chromosome; der = derivative chromosome.

Prior to chemotherapy treatment banding cytogenetics revealed a karyotype 46,XX,del(5q)[8]/46,XX,del(5q),der(17)t(4;17)[5]/45,XX,der(2)t(2;11),der(4)t(4;7),del(5q),-7,der(11)t(11;7;22),der(17)t(4;17),der(22)t(11;22)[9]/46,XX[1] (Figure 1) which was further specified by molecular cytogenetic studies (Figures 2 and 3). Dual-color FISH using a probe specific for BCR and ABL revealed two signals of ABL on both normal chromosome 9, one BCR signal was located on chromosome 22 and the other BCR gene was observed on a der(11) (Figure 2A). Three-color FISH using BCR and ABL mixed with MLL probes revealed the MLL gene signal was located on the short arm of der(11), the other MLL gene signal was observed on der(22), BCR gene signal was located on der(22) and the two ABL gene signals were on the both normal chromosome 9 (Figure 2B). Dual-color FISH using WCP and CEP-specific probes were performed to confirm the rearrangement (data not shown). The locus-specific probe 17p13 (p53) confirmed the presence of TP53 on the normal position in short arm of chromosome 17 (data not shown). Finally, aMCB using probes for the corresponding chromosomes was performed as previously reported [11] (Figure 3). Thus, the following final karyotype was determined:

46,XX,del(5)(q14q34)[8]/46,XX,del(5)(q14q34),der(17)t(4;17)(p13;p13)[5]/45,XX,del(2)(p23),der(4)t(4;7)(p13;q11.23),del(5)(q14q34),-7,der(11)(11qter->11p11.2::11p11.2->11q23::2p23->2pter),der(11)(11pter->11q13::22q11.2->22q13.3::11q13->11q21::7p12->7pter),der(17)t(4;17)(p13;p13),der(22)t(11;22)(q23;q11.2)[9]/46,XX[1].

The abnormal cell population (57%) showed the following immunophenotype: CD45^+dim^(90.4%), HLADr^+^(86%), CD117^+^(57%), CD34^+^(57%), CD18^+^(60%), CD38^+^(83%) and expressed CD2 (50%), CD7(24.2%), CD13 (39%), CD33 (20%), CD123 (65%), CD15 (44%) and CD11c (52%) heterogeneously. The abnormal cells negatively reacted with antibodies to CD10, CD64, CD14, CD16, CD5 and CD19. This immunophenotype was consistent with AML-M4 according to FAB classifications.

## Conclusions

We described a primary AML-M4 case with cytogenetic rearrangements involving seven different chromosomes. According to the literature, not a single case of AML showed a der(4)t(4;7)(p13;q11.23), a der(11)(11qter->11p11.2::11p11.2->11q23::2p23->2pter), a der(17)t(4;17)(p13;p13), or a der(11)(11pter->11q13::22q11.2->22q13.3::11q13->11q21::7p12->7pter) [12]. However, a t(2;11)(p23;q23) was observed in one case of refractory anemia with excess blasts-1 [12]. To the best of our knowledge, the present case is the only one ever seen case of AML with these cytogenetic aberrations [12].

The common chromosomal abnormalities in the AML-M4 include monosomy 5 or del(5q), monosomy 7 or del(7q), trisomy 8, t(6;9) (p23;q34), and rearrangements involving the MLL gene mapped at 11q23 [del(11)(q23); t(9;11)(p22;q23), t(11;19)(q23;p13)], and Core Binding Factor B (CBFβ) mapped at 16q22 [del(16)(q22), inv(16)(p13q22), t(16;16)(p13;q22)] [13]. However, in the present case both MLL genes were intact.

In general, a complex karyotype in MDS or AML is associated with a median survival of less than 1 year [11,14]. Furthermore, the adverse prognostic effect of monosomal karyotype was evident both in the presence and absence of monosomy 5 and/or 7, which suggests that tumor suppressor or other critical genes are not necessarily clustered in specific chromosomes but are instead distributed across several chromosomes [15].

Monosomy 7 is a valuable prognostic marker in AML, and chromosome 7 defects are prominent cytogenetic lesions in primary myelofibrosis, associated with unfavorable prognosis; they present with high incidences after leukemic transformation [16]. Similarly, deletions on 7p12 of *IKZF1* gene (which encodes the transcription factor Ikaros) are associated with a very poor outcome and high relapse rate in B-cell acute lymphocytic leukemia [17]. Monosomy 7 is known as a recurrent cytogenetic aberration in approximately 10% of adult and 5% of childhood AML cases [18]. Jäger et al. [19] found two of seven myeloproliferative neoplasms patients with loss of IKZF1 had monosomy 7. This result suggests that IKZF1 may represent an important tumor-suppressor gene affected by monosomy 7 [19].

The International Prognostic Scoring System (IPSS) classifies cytogenetic and molecular genetic data in AML with clinical data into four risk groups: favorable, intermediate-I, intermediate-II and adverse [20]. The adverse prognostic groups included inv(3)(q21q26.2) or t(3;3)(q21;q26.2); RPN1-EVI1; t(6;9)(p23;q34); DEK-NUP214; t(v;11)(v;q23); MLL rearranged; -5 or del(5q); -7; abnl(17p); complex karyotype [20].

Complex karyotypes, which occur in 10-12% of AML patients, have consistently been associated with a very poor outcome [21]. A complex karyotype has been defined as the presence of 3 or more (in some studies ≥ 5) chromosome abnormalities. For AML it turned out that the presence of t(8;21), inv(16) or t(16;16), and t(15;17) ameliorates the adverse effect of increase karyotypic complexity [20]. As indicated in the new WHO classification, cases with other recurring genetic abnormalities, such as t(9;11) or t(v;11), inv(3) or t(3;3), and t(6;9) should also be excluded from complex rearranged karyotype patient group [22], because these groups constitute separate entities. One striking observation is the increasing incidence of adverse versus favorable cytogenetic abnormalities with increasing age. This, at least in part, contributes to the poorer outcome of AML in older adults [23].

In conclusion, we reported a de novo case of AML-M4 with yet unreported translocation events involving seven different chromosomes. Taken together all findings an adverse prognosis for this specific AML-case must be considered.

## Materials and methods

### Case report

A 65-year-old woman was diagnosed as suffering from AML in September 2011. Anemia, thrombocytopenia, fever, fatigue and weight loss were the indicative symptoms. Her hematologic parameters were: white blood cells (WBC) of 34.2×10^9^/l with 25.5% neutrophils, 36.2% lymphocytes, and 38.3% immature cells, red blood cell (RBC) count was 1.86×10^6^/mm^3^, hemoglobin level was 6.7 g/dl and the platelet count was 19×10^9^/l. No treatment had been administered prior to the tests mentioned below. All human studies have been approved by the ethics committee of the Atomic Energy Commission, Damascus, Syria and have therefore been performed in accordance with the ethical standards laid down in the 1964 Declaration of Helsinki and its later amendments. The patient gave his informed consent prior to its inclusion in this study. Later the patient was lost during follow-up.

### Chromosome analysis

Chromosome analysis using GTG-banding was performed according to standard procedures [24]. A minimum of 20 metaphase cells derived from unstimulated bone marrow culture were analyzed. Karyotypes were described according to the International System for Human Cytogenetic Nomenclature [25].

### Molecular cytogenetics

Fluorescence in situ hybridization (FISH) using LSI BCR/ABL dual color dual fusion translocation probe (Abbott Molecular/Vysis, Des Plaines, IL, USA), MLL break-apart probe (Q-Biogene, USA) mixed with LSI BCR/ABL dual color dual fusion translocation probe chromosome enumeration probe (CEP) for chromosomes 9 and 11 (Abbott Molecular /Vysis) and 17p13 (p53), dual color probe (Q-Biogene, USA) were applied according to manufacturer’s instructions. Whole chromosome painting (WCP) probes for chromosomes 2, 4, 5, 7, 11, 17 and 22 were also applied (MetaSystems, Altlussheim, Germany) [24]. FISH using the corresponding chromosome specific array-proven multicolor banding (aMCB) probe sets based on microdissection derived region-specific libraries was performed as previously reported [26]. A minimum of 20 metaphase spreads were analyzed, using a fluorescence microscope (AxioImager.Z1 mot, Carl Zeiss Ltd., Hertfordshir, UK) equipped with appropriate filter sets to discriminate between a maximum of five fluorochromes plus the counterstain DAPI (4′,6- diamino-2-phenylindole). Image capture and processing were performed using an ISIS imaging system (MetaSystems).

### Flow cytometric immunophenotype

Flow cytometric analysis was performed using a general panel of fluorescent antibodies against the following antigens typical for different cell lineages and cell types: CD1a, CD2, CD3, CD4, CD5, CD8, CD10, CD11b, CD11c, CD13, CD14, CD15, CD16, CD19, CD20, CD22, CD23, CD32, CD33, CD34, CD38, CD41a, CD45, CD56, CD57, CD64, CD103, CD117, CD123, CD138, CD209, CD235a and CD243; In addition to antibodies to Kappa and Lambda light Chains, IgD, sIgM, and HLADr. All antibodies purchased from BD Biosciences. Samples analyzed on a BD FACSCalibur™ flow cytometer. Autofluorescence, viability, and isotype controls were included. Flow cytometric data acquisition and analysis were conducted by BD Cellquest™ Pro software.

## Competing interests

The authors declare that they have no competing interests.

## Authors’ contributions

WA-A, AA and AW provided the case and/or did primary cytogenetic and main part of the FISH-tests; MAKO did detailed FISH studies. WA drafted the paper and all authors read and approved the final manuscript.
